# Procedural and post-operative complications associated with laparoscopic versus open abdominal surgery for right-sided colonic cancer resection: A systematic review and meta-analysis

**DOI:** 10.1097/MD.0000000000022431

**Published:** 2020-10-02

**Authors:** Yong Sheng Li, Fan Chun Meng, Jun Kai Lin

**Affiliations:** a Department of Colorectal surgery; b Department of Gastroenterology, Dongying Shengli Oilfield Central Hospital, Dongying, Shandong, P.R. China.

**Keywords:** anastomotic leak, colon cancer, hospital stay, ileus, laparoscopic surgery, open surgery, post-operative complications, risk ratios

## Abstract

**Background:**

In this analysis, we aimed to systematically compare the procedural and post-operative complications (POC) associated with laparoscopic versus open abdominal surgery for right-sided colonic cancer resection.

**Methods:**

We searched MEDLINE, http://www.ClinicalTrials.gov, EMBASE, Web of Science, Cochrane Central, and Google scholar for English studies comparing the POC in patients who underwent laparoscopic versus open surgery (OS) for right colonic cancer. Data were assessed by the Cochrane-based RevMan 5.4 software (The Cochrane Community, London, UK). Mean difference (MD) with 95% confidence intervals (CIs) were used to represent the results for continuous variables, whereas risk ratios (RR) with 95% CIs were used for dichotomous data.

**Results:**

Twenty-six studies involving a total number of 3410 participants with right colonic carcinoma were included in this analysis. One thousand five hundred and fifteen participants were assigned to undergo invasive laparoscopic surgery whereas 1895 participants were assigned to the open abdominal surgery. Our results showed that the open resection was associated with a shorter length of surgery (MD: 48.63, 95% CI: 30.15–67.12; *P* = .00001) whereas laparoscopic intervention was associated with a shorter hospital stay [MD (–3.09), 95% CI [–5.82 to (–0.37)]; *P* = .03]. In addition, POC such as anastomotic leak (RR: 0.96, 95% CI: 0.60–1.55; *P* = .88), abdominal abscess (RR: 1.13, 95% CI: 0.52–2.49; *P* = .75), pulmonary embolism (RR: 0.40, 95% CI: 0.09–1.69; *P* = .21) and deep vein thrombosis (RR: 0.94, 95% CI: 0.39–2.28; *P* = .89) were not significantly different. Paralytic ileus (RR: 0.87, 95% CI: 0.67–1.11; *P* = .26), intra-abdominal infection (RR: 0.82, 95% CI: 0.15–4.48; *P* = .82), pulmonary complications (RR: 0.83, 95% CI: 0.57–1.20; *P* = .32), cardiac complications (RR: 0.73, 95% CI: 0.42–1.27; *P* = .27) and urological complications (RR: 0.83, 95% CI: 0.52–1.33; *P* = .44) were also similarly manifested. Our analysis also showed 30-day re-admission and re-operation, and mortality to be similar between laparoscopic versus OS for right colonic carcinoma resection. However, surgical wound infection (RR: 0.65, 95% CI: 0.50–0.86; *P* = .002) was significantly higher with the OS.

**Conclusions:**

In conclusion, laparoscopic surgery was almost comparable to OS in terms of post-operative outcomes for right-sided colonic cancer resection and was not associated with higher unwanted outcomes. Therefore, laparoscopic intervention should be considered as safe as the open abdominal surgery for right-sided colonic cancer resection, with a decreased hospital stay.

## 1 Introduction

Colon cancer is among the most common cancers occurring in both men and women and resection is the only way to cure this condition.^[1]^ Previously, large abdominal incisions were carried out to remove colon cancers. However, advance in medical technology has made laparoscopic resection possible. Laparoscopic colectomy was first introduced in the year 1991^[2]^ and soon after, it became a better option for patients with colon cancers who required surgical intervention.

Even though laparoscopic colon resection has well been accepted for the treatment of left and transverse colon cancer, this was not the case with right colon cancer. In fact, due to the complexity of right colon laparoscopic anatomy and variable vascular peduncles that might require a greater laparoscopic experience than left colon and rectum surgery, many surgeons considered laparoscopic approach to right colon a useless and a complete waste of time.^[3]^ However, fortunately different laparoscopic hybrid procedures including total laparoscopic right colectomy,^[4]^ single incision laparoscopic surgery (LS) for right colon,^[5]^ laparoscopic assisted right colectomy,^[6]^ hand-assisted right colectomy with laparoscopic mobilization of colon by hand to the right side,^[7]^ which have been developed to facilitate the intervention for right colonic cancers.

An editorial proved that laparoscopic right hemicolectomy for colon cancer is technically feasible and safe to be carried out in terms of oncological outcomes.^[8]^ However, because the study included outcomes of a single surgery, the author stated that further studies with a larger sample size might be able to better prove the significance of laparoscopic right hemicolectomy.

In this analysis, we aimed to systematically compare the procedural and post-operative complications (POC) associated with laparoscopic versus open abdominal surgery for right-sided colonic cancer resection.

## 2 Methods

### 2.1 Search databases and search strategies

We searched MEDLINE, http://www.ClinicalTrials.gov, EMBASE, Web of Science, Cochrane Central, and Google scholar for English studies comparing the POC in patients who underwent laparoscopic versus open abdominal surgery for right colonic cancer.

We used the following searched terms and phrases during this database searched process:

- laparoscopic versus open surgery (OS) for colon cancer;
- laparoscopic versus OS for right colon cancer;
- laparoscopic versus OS for right colonic carcinoma;
- laparoscopic versus open AND right colon cancer;
- invasive versus open abdominal surgery for colon cancer.

### 2.2 Inclusion and exclusion criteria

Inclusion criteria consisted of studies that:

- compared laparoscopic versus open abdominal surgery for right colonic carcinoma;
- reported POC following surgical interventions;
- were published in English language.

Exclusion criteria consisted of studies that:

- did not compare laparoscopic versus open abdominal surgery for right colonic carcinoma;
- involved other colon carcinoma apart from right colonic cancers;
- did not report any POC;
- were published in a different language apart from English;
- consisted only of an abstract; the full-text article was not available;
- were repeated studies which were obtained from different search databases.

### 2.3 Endpoints to be assessed in this analysis

Table 1 lists the post-operative outcomes which were reported in the original studies.

**Table 1:**
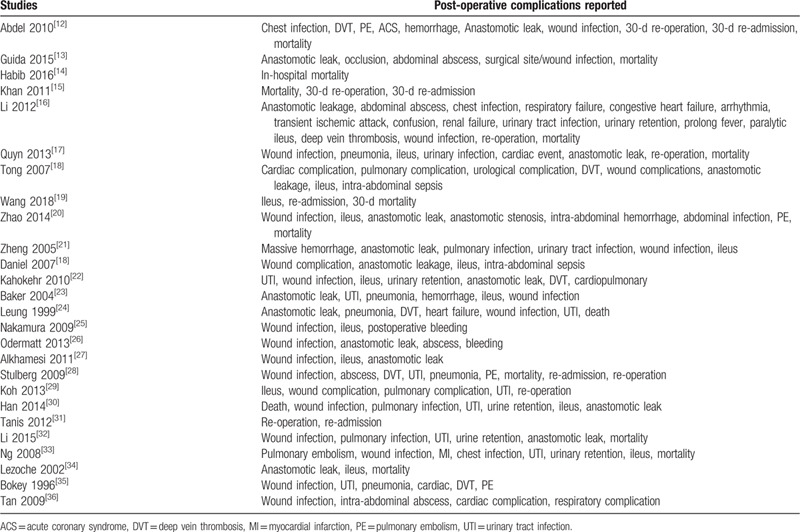
Complications which were reported in the previous studies.

The endpoints which were assessed in this analysis included:

(a) Duration time period of surgical intervention;
(b) length of hospital stay;
(c) anastomotic leak;
(d) surgical wound infection;
(e) abdominal abscess;
(f) pulmonary embolism;
(g) deep vein thrombosis;
(h) paralytic ileus;
(i) intra-abdominal infection;
(j) pulmonary complications involving complications related to the lungs such as pulmonary embolism, chest infection, respiratory failure;
(k) cardiac complications involving complications related to the heart such as acute coronary syndrome, arrhythmia, heart failure;
(l) urological complications including conditions such as urinary tract infections, and urinary retention;
(m) 30-day re-admission;
(n) 30-day re-operation;
(o) mortality.

### 2.4 Data extraction and quality assessment

Data including the duration time period of the surgical intervention, the length of hospital stay, the number of events reported for POC, the type of study, the participants’ enrollment time period, the total number of participants who were assigned to the laparoscopic and open abdominal surgical interventions respectively, the baseline characteristics including the median age, the percentage of male participants, the body mass index value, and features describing the methodological quality of the studies were carefully extracted by 3 independent authors.

Any disagreement was referred to the corresponding author for further consideration. It was the responsibility of the corresponding author to take the final decision.

The Newcastle Ottawa Scale^[9]^ and the Cochrane collaboration^[10]^ were the tools used to assess the methodological quality of the studies for observational cohorts and randomized trials respectively. Grades (A, B, or C) representing low, moderate and high risk of bias was then allotted to the respective studies.

### 2.5 Statistical analysis

Throughout the analysis, the Cochrane-based RevMan 5.4 software (The Cochrane Community, London, UK) was used to assess the data.

Since continuous data (mean and standard deviation) were used to report for the duration time period of surgery and the mean length of hospital stay, mean difference (MD) with 95% confidence intervals (CIs) were used to represent the results.

Dichotomous data were used to report the POC. Therefore, risk ratios (RR) with 95% CIs were used to represent the results.

Heterogeneity was assessed by the Q statistic test. Results representing a *P* value equals to or less than .05 were considered statistically significant. The I^2^ statistic test was also used to assess for heterogeneity. The larger the I^2^ value, the larger the heterogeneity. In addition, based on this heterogeneity value, either a fixed or a random statistic model was applied respectively.

Sensitivity analysis was carried out by excluding each of the studies, 1 at a time and a new analysis was carried out each time and was compared with the main results of this study. Also, publication bias was estimated through a visual assessment of the funnel plots.

### 2.6 Ethical approval

Ethical approval or compliance with ethical guidelines was not required for systematic reviews and meta-analyses.

## 3 Results

### 3.1 Search database outcomes

Our search resulted with over 5500 articles (5802 more precisely). The PRISMA reporting guideline was used.^[11]^ The 3 authors carefully assessed the titles to see if they matched with the scope of this research paper. If title of the publications were irrelevant, they were directly eliminated (4128). The authors also carefully assessed the abstracts of the remaining articles (1674). The abstracts were checked for relevant data and outcomes. Any abstract that did not report the relevant data or outcomes were eliminated (1318).

Only, 356 full text articles were assessed for eligibility. Further eliminations were carried out based on the inclusion and exclusion criteria:

Literature review (9), systematic reviews (8), meta-analyses (28), letters of correspondence (7), case studies (31), compared laparoscopic versus open abdominal surgery of left or transverse colon (109), did not report the POC (19), involved rectal carcinoma (44), published in another language (8), full-text was not available (9), repeated studies (58).

**Figure 1:**
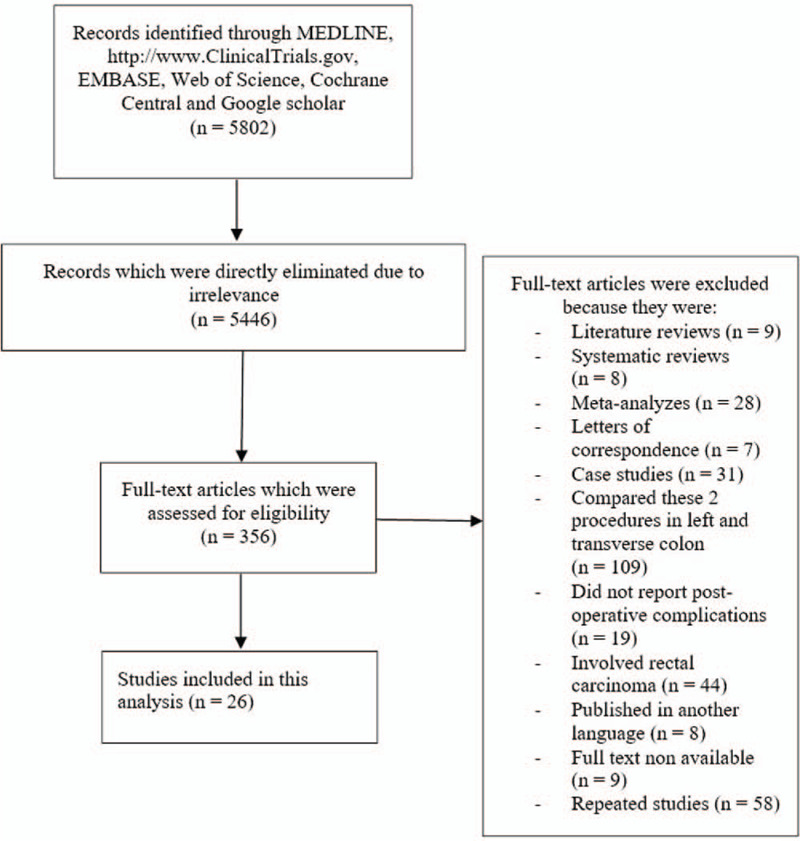
Flow diagram showing the study selection.

Finally only 26 studies[^12^,^13^,^14^,^15^,^16^,^17^,^18^,^19^,^20^,^21^,^22^,^23^,^24^,^25^,^26^,^27^,^28^,^29^,^30^,^31^,^32^,^33^,^34^,^35^,^36^] were included in this meta-analysis. The flow diagram for the study selection has been shown in Figure 1.

### 3.2 Characteristics of the studies and participants

Twenty-six studies involving a total number of 3410 participants with right colonic cancer were included in this analysis. One thousand five hundred and fifteen participants were assigned to undergo invasive LS whereas 1895 participants were assigned to the open abdominal surgery as shown in Table 2. The participants were enrolled between the years 1992 to 2017. Most of the studies were observational cohorts.

**Table 2:**
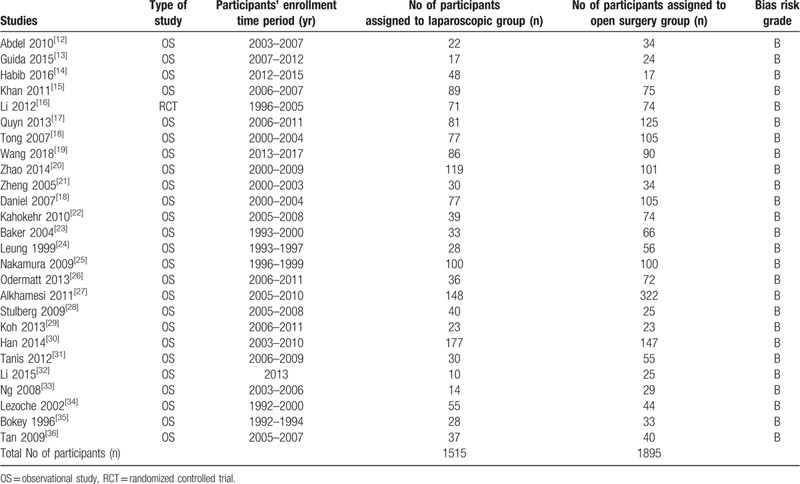
General features of the studies.

The studies were allotted a bias grade B denoting moderate risk following a methodological assessment.

Table 3 lists the baseline features as well as the number of days of hospital stay.

**Table 3:**
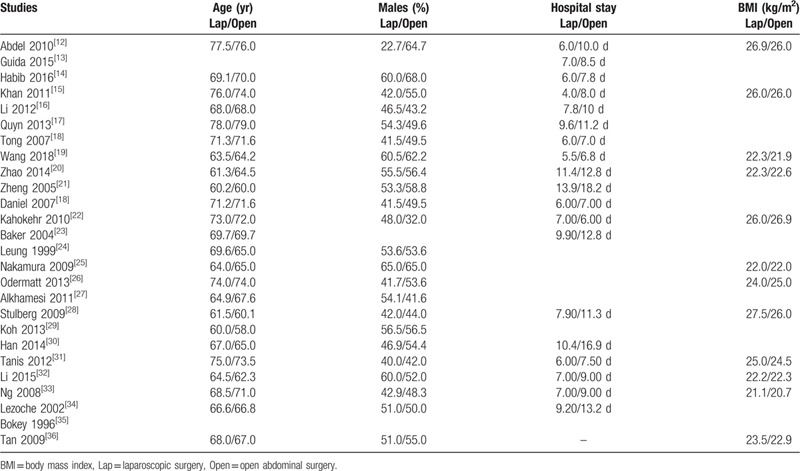
Baseline features of the participants.

### 3.3 Analysis of the procedural length and POC associated with laparoscopic versus OS for right-sided colon carcinoma

**Figure 2:**
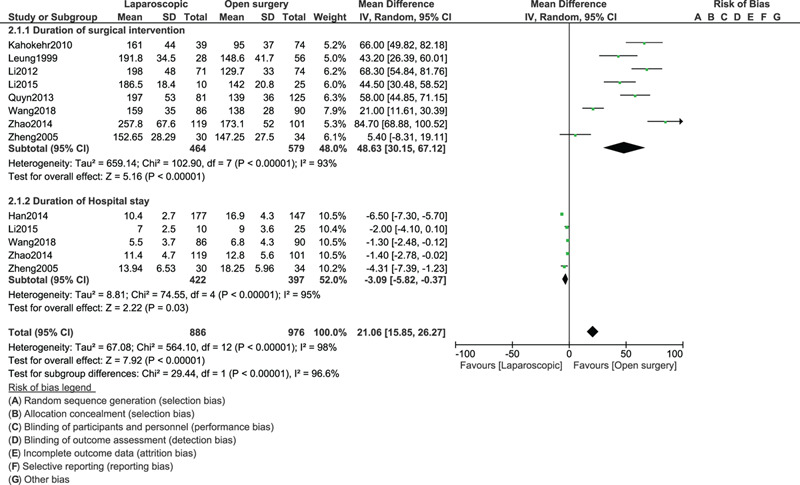
Length duration of surgical intervention and hospital stay between laparoscopic and open abdominal surgery for right colon cancer.

Our results showed that the OS for right-sided colonic cancer was associated with a shorter length of surgery (MD: 48.63, 95% CI: 30.15–67.12; *P* = .00001) as shown in Figure 2. However, laparoscopic intervention was associated with a shorter hospital stay [MD (–3.09), 95% CI [–5.82 to (–0.37)]; *P* = .03] as shown in Figure 2.

**Figure 3:**
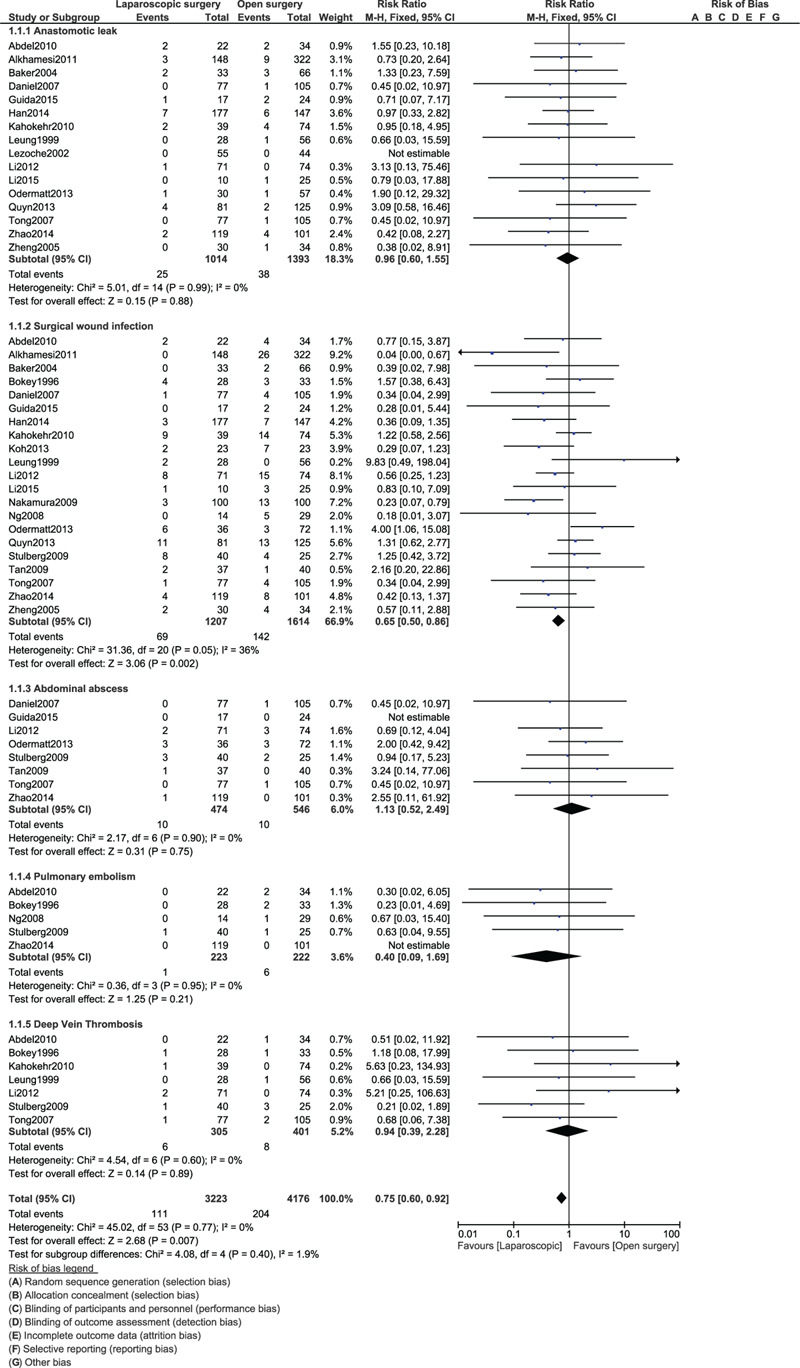
Post-operative outcomes between laparoscopic versus open surgery for right colon cancer (part I).

When the POC were compared between laparoscopic versus OS for right colonic cancer resection, anastomotic leak (RR: 0.96, 95% CI: 0.60–1.55; *P* = .88), abdominal abscess (RR: 1.13, 95% CI: 0.52–2.49; *P* = .75), pulmonary embolism (RR: 0.40, 95% CI: 0.09–1.69; *P* = .21) and deep vein thrombosis (RR: 0.94, 95% CI: 0.39–2.28; *P* = .89) were not significantly different as shown in Figure 3. However, surgical wound infection (RR: 0.65, 95% CI: 0.50–0.86; *P* = .002) was significantly higher with the OS.

**Figure 4:**
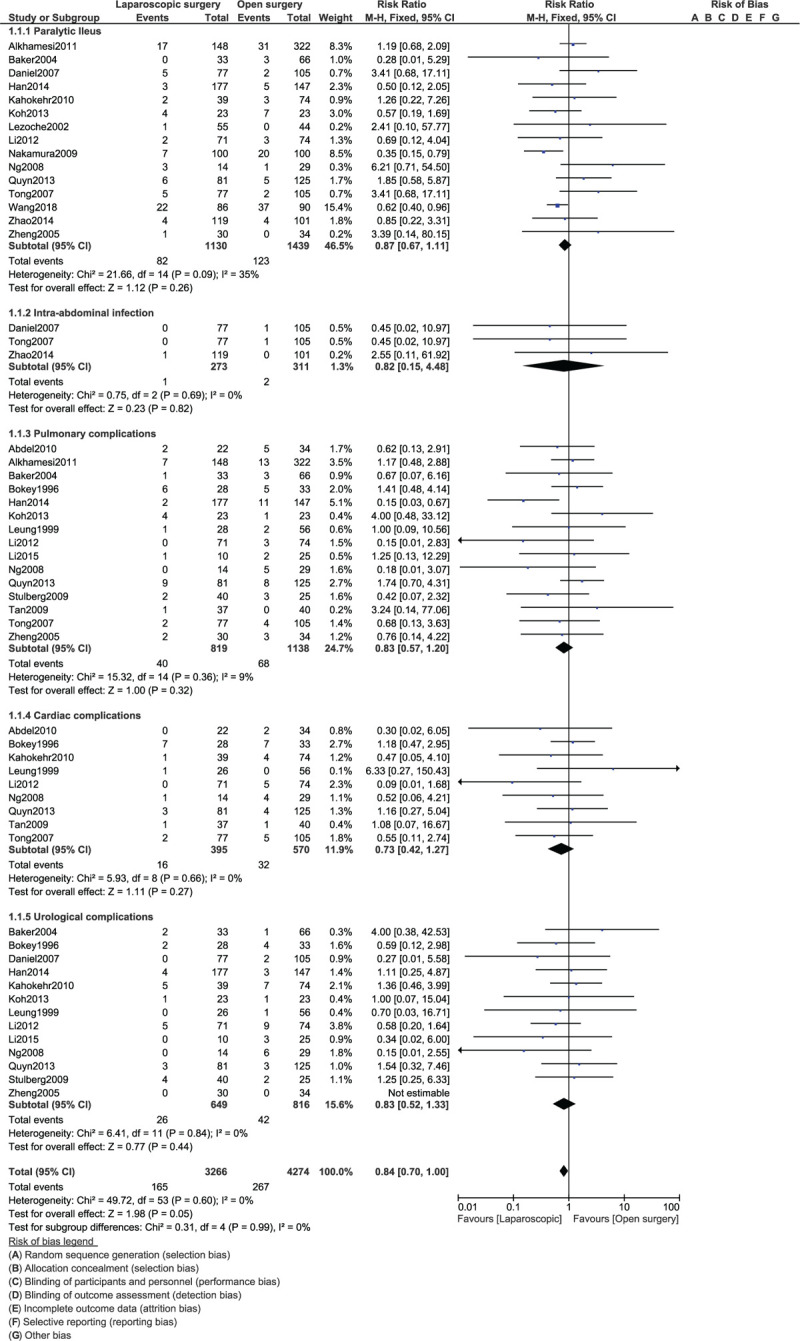
Post-operative outcomes between laparoscopic versus open surgery for right colon cancer (part II).

Paralytic ileus (RR: 0.87, 95% CI: 0.67–1.11; *P* = .26), intra-abdominal infection (RR: 0.82, 95% CI: 0.15–4.48; *P* = .82), pulmonary complications (RR: 0.83, 95% CI: 0.57–1.20; *P* = .32), cardiac complications (RR: 0.73, 95% CI: 0.42–1.27; *P* = .27) and urological complications (RR: 0.83, 95% CI: 0.52–1.33; *P* = .44) were similarly manifested as shown in Figure 4.

**Figure 5:**
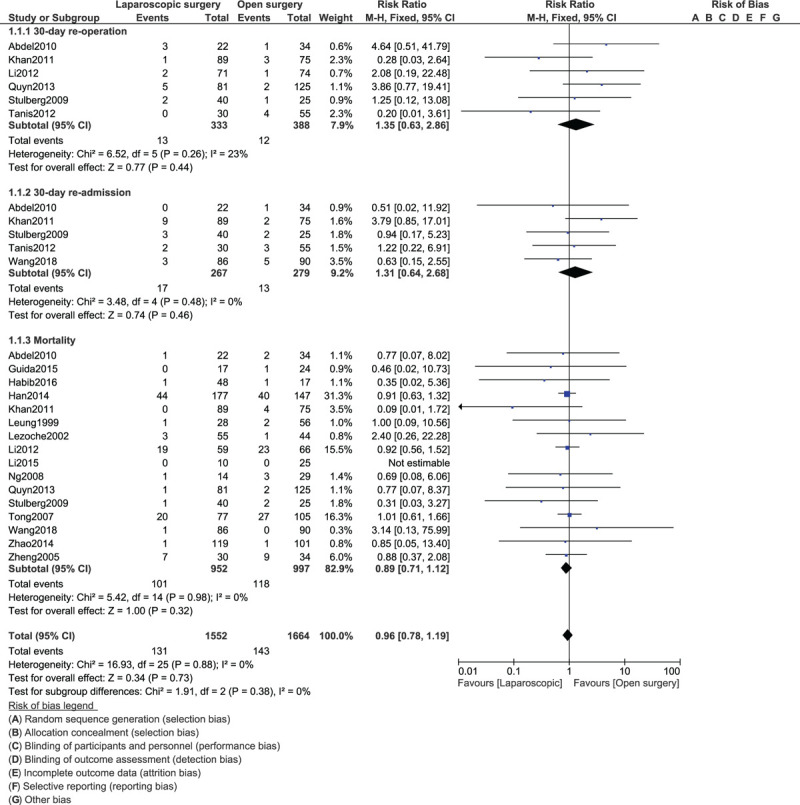
Post-operative outcomes between laparoscopic versus open surgery for right colon cancer (part III).

Our analysis also showed 30-day re-admission (RR: 1.31, 95% CI: 0.64–2.68; *P* = .46), 30-day re-operation (RR: 1.35, 95% CI: 0.63–2.86; *P* = .44), and mortality (RR: 0.89, 95% CI: 0.71–1.12; *P* = .32) to be similar between laparoscopic versus OS for right colonic cancer resection as shown in Figure 5.

The results were summarized in Table 4.

**Table 4:**
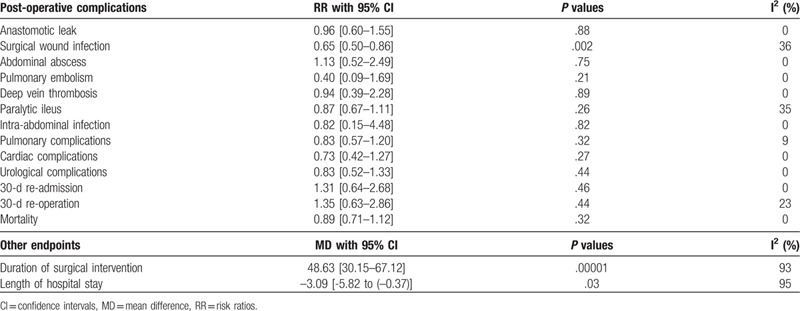
Main results of this analysis.

### 3.4 A subgroup analysis of participants who underwent emergency surgery for right-sided colonic cancer resection

**Figure 6:**
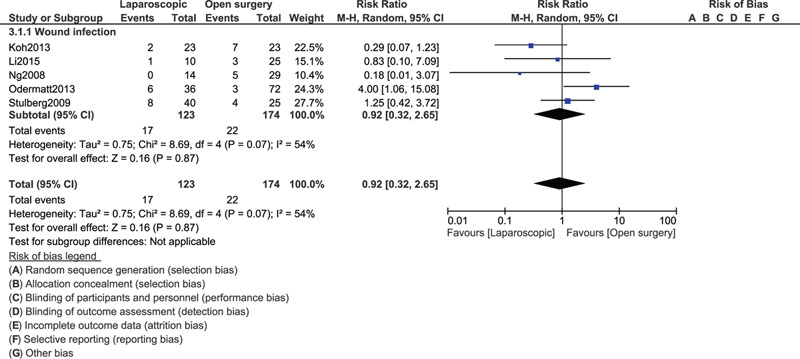
Post-operative outcomes for emergency laparoscopic versus open surgery for right colon cancer (part I).

**Figure 7:**
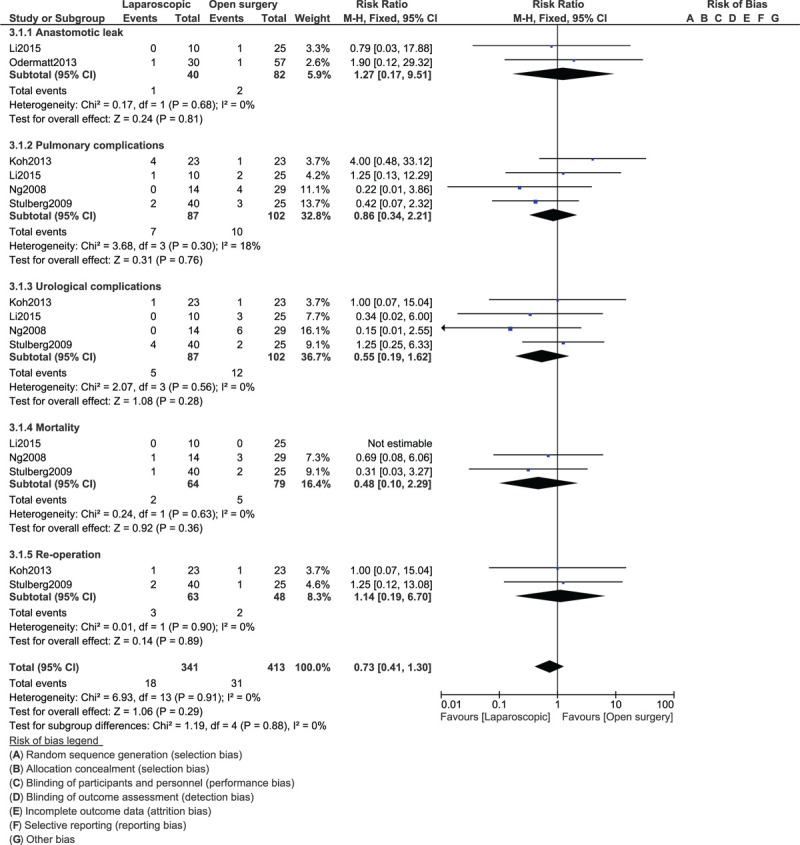
Post-operative outcomes for emergency laparoscopic versus open surgery for right colon cancer (part II).

We also carried out a subgroup analysis showing the POC associated with emergency laparoscopic versus OS for right-sided colon carcinoma. Our results showed that wound infection (RR: 0.92, 95% CI: 0.32–2.65; *P* = .87) was similar in both groups as shown in Figure 6. In addition, anastomotic leak (RR: 1.27, 95% CI: 0.17–9.51; *P* = .81), pulmonary complications (RR: 0.86, 95% CI: 0.34–2.21; *P* = .76), urological complications (RR: 0.55, 95% CI: 0.19–1.62; *P* = .28), mortality (RR: 0.48, 95% CI: 0.10–2.29; *P* = .36) and re-operation (RR: 1.14, 95% CI: 0.19–6.70; *P* = .89) were also similarly manifested as shown in Figure 7.

**Figure 8:**
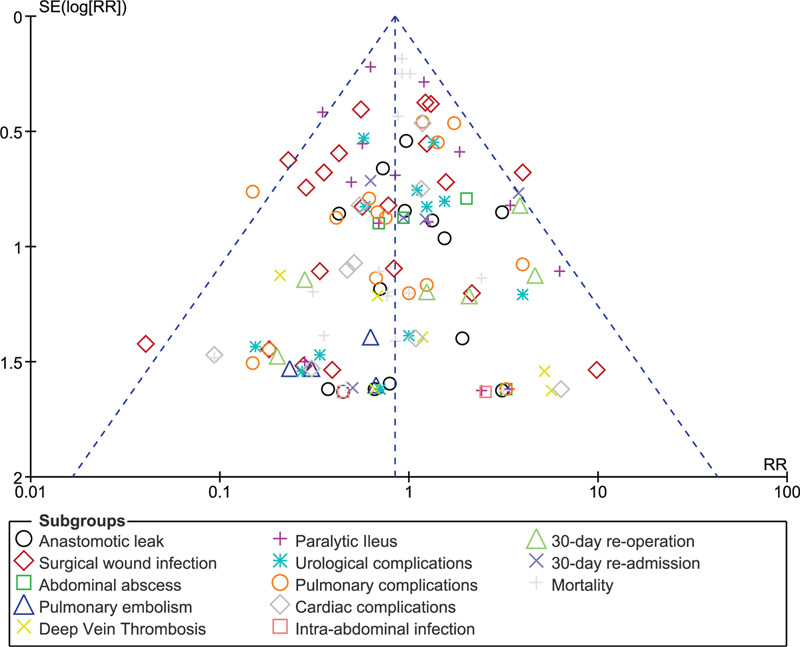
Funnel plot showing publication bias.

Consistent results involving procedural duration time, length of hospital stay, and POC were obtained throughout following sensitivity analyses. No deviation was observed from the main results. In addition, the funnel plot was symmetrical indicating a low evidence of publication bias among the studies which assessed these POC as shown in Figure 8.

## 4 Discussion

Based on the results of this analysis, it was observed that laparoscopic intervention for right colon cancer was equally effective and safe compared to the open abdominal surgery and was not associated with higher POC except for surgical wound infection which was significantly higher in the OS group. Anastomotic leak, abdominal abscess, pulmonary embolism, deep vein thrombosis, paralytic ileus, intra-abdominal infection, other pulmonary, cardiac, and urological complications were similarly observed with both interventions. In addition, the length of hospital stay following LS was shorter compared to the open surgical intervention. However, it was more time consuming compared to the open abdominal surgery.

Pulmonary embolism and deep vein thrombosis have often been common POC especially for those patients who require strict bed rest for a longer duration of time post operatively. LS is associated with a short hospital stay which could allow the patient to better mobilize sooner after the surgery, and could be a better advantage to reduce the risk of any pulmonary embolism^[37]^ or deep vein thrombosis.^[38]^ In addition, smaller abdominal incisions are done in laparoscopic surgeries which would allow a rapid healing time, and lesser chances for surgical wound infections^[39]^ when compared to the open abdominal surgeries for right-sided colon carcinoma.

A recent systematic review and meta-analysis showed LS to have similar intraoperative and postoperative recovery parameters compared to the open surgical procedure.^[40]^ The analysis even showed that duration of surgery was longer with the laparoscopic technique. However, advantages included a shorter hospital stay, minimal intraoperative blood loss, and shorter length of incision.

Another systematic review and meta-analysis showed LS to be associated with a similar survival rate compared to the OS again supporting the results of this current analysis.^[41]^ However, the authors stated that OS was associated with more harvest of affected lymph nodes but they are not sure whether this was clinically significant. The authors also stated that surgeons should always be prepared for the conversion of laparoscopic to open abdominal surgery.

Our analysis was based on patients with right colon cancer. A retrospective cohort study^[42]^ using data identified from the Ontario Cancer Registry and physician billing data between January 2010 and December 2014 showed that patients who underwent LS were most likely to be from urban areas, and have undergone planned surgeries, and to have minimal local tumor invasions compared to those undergoing OS. However, there was no significant difference in post discharge symptoms. In addition, other systematic reviews and meta-analyses have also been published.[^43^,^44^]

Even though this current analysis showed no significant difference in POC between laparoscopic resection versus OS for right colon cancers, another study,^[30]^ which aimed to investigate the applicability, safety, short term, and long term outcomes of laparoscopic versus open resection for the treatment of right colon cancer with D3 lymphadenectomy, showed that even if both operative techniques were effective and safe, the laparoscopic-assisted right hemicolectomy with D3 lymphadenectomy was also superior in terms of short term outcomes. Also, several developments are continually being done for the management of right-sided colonic disease including robotic right hemicolectomy which apparently could show positive outcomes.^[45]^ However, it would be vital to also consider the costs of these new robotic, laparoscopic and open abdominal surgeries.[^46^,^47^,^48^]

### 4.1 Limitations

We have described the limitations as follow: Due to the inclusion of a total number of only 3410 participants, the results might have to be confirmed in larger studies with far more participants. Another limitation could be the fact that the co-morbidities prior to surgery was ignored. Moreover, many endpoints were not reported in all the original studies, and therefore, several subgroups assessing different POC included only a minimum number of studies which could be another limitation of this analysis. Another limitation could be the fact that most of the studies which were included in this analysis were observation cohorts (90%). The original studies were researches carried out in different hospitals from different parts of the world with differences in hospital settings and peri-operative care. This might have had an impact on the outcomes. At last, nowadays there are immense improvements in operative techniques, operative equipment and hospital operative settings when compared to previous years. This variation in previous and recent hospital set ups and improved technologies might also be another limitation of this analysis.

## 5 Conclusions

In conclusion, LS was almost comparable to OS in terms of post-operative outcomes for right-sided colonic cancer resection and was not associated with higher unwanted outcomes. Therefore, laparoscopic intervention should be considered as safe as the open abdominal surgery for right-sided colonic cancer resection, with a decreased hospital stay.

## Author contributions

The authors Yong Sheng Li, Fan Chun Meng and Jun Kai Lin were responsible for the conception and design, acquisition of data, analysis and interpretation of data, drafting the initial manuscript, and revising it critically for important intellectual content. Yong Sheng Li and Fan Chun Meng are the first co-authors and they wrote this manuscript, agreed and approved it as it is.

**Conceptualization:** Yong Sheng Li, Fan Chun Meng, Jun Kai Lin.

**Data curation:** Yong Sheng Li, Fan Chun Meng, Jun Kai Lin.

**Formal analysis:** Yong Sheng Li, Fan Chun Meng, Jun Kai Lin.

**Funding acquisition:** Yong Sheng Li, Fan Chun Meng, Jun Kai Lin.

**Investigation:** Yong Sheng Li, Fan Chun Meng, Jun Kai Lin.

**Methodology:** Yong Sheng Li, Fan Chun Meng, Jun Kai Lin.

**Project administration:** Yong Sheng Li, Fan Chun Meng, Jun Kai Lin.

**Resources:** Yong Sheng Li, Fan Chun Meng, Jun Kai Lin.

**Software:** Yong Sheng Li, Fan Chun Meng, Jun Kai Lin.

**Supervision:** Yong Sheng Li, Fan Chun Meng, Jun Kai Lin.

**Validation:** Yong Sheng Li, Fan Chun Meng, Jun Kai Lin.

**Visualization:** Yong Sheng Li, Fan Chun Meng, Jun Kai Lin.

**Writing – original draft:** Yong Sheng Li, Fan Chun Meng.

**Writing – review & editing:** Yong Sheng Li, Fan Chun Meng.
